# Microscopic and Mechanical Characterization of Co-Cr Dental Alloys Joined by the TIG Welding Process

**DOI:** 10.3390/ma16103890

**Published:** 2023-05-22

**Authors:** Andreja Carek, Ljerka Slokar Benić, Vatroslav Bubalo, Nika Kosović

**Affiliations:** 1 School of Dental Medicine, University of Zagreb, Ivana Gundulića 5, 10000 Zagreb, Croatia; acarek@sfzg.unizg.hr (A.C.); nkosovic@sfzg.unizg.hr (N.K.); 2 Faculty of Metallurgy, University of Zagreb, Aleja Narodnih Heroja 3, 44000 Sisak, Croatia; 3 Dubrava University Hospital, Avenija Gojka Šuška 6, 10000 Zagreb, Croatia; vat.bub@inet.hr

**Keywords:** microstructure, mechanical properties, cobalt-chromium dental alloys, microhardness, flexural strength, dynamic testing, TIG welding

## Abstract

Due to their good mechanical and other properties, cobalt-chromium alloys (Co-Cr) are often used in prosthetic therapy. The metal structures of prosthetic works can be damaged and break, and depending on the extent of the damage, they can be re-joined. Tungsten inert gas welding (TIG) produces a high-quality weld with a composition very close to that of the base material. Therefore, in this work, six commercially available Co-Cr dental alloys were joined by TIG welding, and their mechanical properties were evaluated to determine the quality of the TIG process as a technology for joining metallic dental materials and the suitability of the Co-Cr alloys used for TIG welding. Microscopic observations were made for this purpose. Microhardness was measured using the Vickers method. The flexural strength was determined on a mechanical testing machine. The dynamic tests were carried out on a universal testing machine. The mechanical properties were determined for welded and non-welded specimens, and the results were statistically evaluated. The results show the correlation between the investigated mechanical properties and the process TIG. Indeed, characteristics of the welds have an effect on the measured properties. Considering all the results obtained, the TIG—welded I—BOND NF and Wisil M alloys showed the cleanest and most uniform weld and, accordingly, satisfactory mechanical properties, highlighting that they withstood the maximum number of cycles under dynamic load.

## 1. Introduction

The mechanical properties of dental alloys, which result from the chemical composition and structure of the casting material, are crucial for the technical and functional durability of prosthetic therapy. Important mechanical properties are: hardness, strength, elasticity and elongation. In the clinic, it is very difficult to find materials that have all these favorable properties at the same time, so compromises often have to be made. The materials must have optimal strength and stiffness so that they can withstand the masticatory load without cracking and breaking and so that they can retain their original shape. A list of the different dental alloys and their mechanical properties is given in Table 1. The most commonly used alloy is the cobalt-chromium alloy due to its good properties such as: high strength, resistance to corrosion, wear, heat and tarnishing and acceptable weldability. Cobalt-chromium alloys, which are predominantly base metals, are often used for fixed works, frameworks for removable partial dentures, etc. [1,2,3]. The classification and mechanical properties of the commonly used Co-Cr dental alloys are listed in Table 2.

The good mechanical properties of these metals are the result of their multiphase structure. In fact, the various components of the alloy are hardened by the aging and precipitation of carbides, which extraordinarily increase their hardness [25,26]. One of the most important constituents of the alloy is carbon, which influences the microhardness and ductility of the alloy; however, together with chromium, it forms chromium carbide, which results in higher hardness and strength but also increases the internal stresses and makes the alloy more brittle [27,28]. However, as the oral cavity is constantly in motion, resistance to strong masticatory forces and exposure to saliva are extremely important. The hardness of the cobalt-chromium alloy is between 550 and 800 MPa, and the tensile strength is between 145 and 270 MPa [29]. Unfortunately, it is common for the metal structures of prosthetic works to be damaged and break, and depending on the extent of the damage, they can be rejoined. For this purpose, one of the joining methods should be used. Welding techniques are becoming increasingly popular due to the better compatibility and strength of the resulting joints, as well as the higher productivity. The most commonly used welding techniques in dentistry are laser welding and tungsten inert gas (TIG) welding [30,31]. Both have certain advantages over other welding techniques. They allow for welding directly on the model and have a smaller heat-affected zone (HAZ) because the energy is concentrated in a small area, which allows for working close to the resin and ceramic. The basic difference between laser welding and TIG welding is that TIG uses electrical energy, while the laser applies light energy to the object. Another difference is the cost of the equipment. Due to the higher cost of laser welders, they are not commonly used in dental laboratories [29,31]. In addition, TIG welding produces a high-quality weld with a composition very close to that of the base material [32].

In the TIG process, a protective gas atmosphere is required to minimize the interaction with the air and maintain an inert atmosphere during work, which is the main cause of porosity in the welding area. Air bubbles are the starting point for fractures and stress points. The choice of TIG parameters is complicated and depends on material thickness, technological requirements, equipment, etc. Most manufacturers now offer automatic setting of the parameters depending on the selected material type and thickness. The surface of the object in the welding area must be as clean as possible and free of grease. Soot can form on poorly cleaned parts. Soot can also be the result of using an incorrect gas or a poor gas setting. When working with TIG, the heat is distributed over a larger area, so the heating surface is larger but also stronger than that with a laser-welded joint. When spot welding, the overlap of the working surface should be between 50 and 70%, i.e., the distance between the welding spots should not be too large [33,34].

The welds of dental alloys used in prosthetics are areas of increased stress concentration and are critical areas for fracture and corrosion instability. Previous research has shown that when comparing laser and TIG welding, TIG welds are much more flexible and exhibit more deformation than laser welds, which are also harder. In a subsequent study monitoring the stress distribution around implants in laser- and TIG-welded screw frames, there was no difference between the techniques, i.e., the stress results were similar for both techniques and independent of the measurement region on the bone [29,35]. In another study, also using cobalt-chromium alloys from different manufacturers, the homogeneity of laser and TIG welds was investigated. The laser weld was clean, with overlaps of 20–40%, but cracks were also visible along the entire length of the weld. In the TIG-welded sample, the weld material is uniform, with no visible irregularities and less porosity [36]. The results also show that some welds have macroscopically insufficient rows of dots and insufficient overlaps, as well as many cracks and pores. It was concluded that solderers and welders need good eyesight, hand-eye coordination and manual dexterity. They should be able to concentrate on detailed work for a long period of time. Cracks were probably due to specimen handling and internal stresses, but the microscopic homogeneity of welds was considered (both TIG and laser and braze welds). Laser-welded alloys often exhibit defects due to residual stresses caused by temperature variations during the welding process. Residual stresses affect the mechanical properties of the weld, so it is safer to work with TIG to avoid microcracks [33,36].

De Castro Silverio et al. evaluated Co-Cr joints under different welding parameters. They showed that the machine parameters are only one aspect in evaluating the success of the welding technique [37]. Bock et al. applied laser and TIG welding on stainless steel for orthodontic purposes and showed that the microhardness and tensile strength of TIG welds were comparable to those of laser welds [32]. A.O. Matos et al. performed a comparative analysis of the ceramic flexural strength of Co-Cr alloys joined by TIG welding and conventional brazing. They showed that the TIG process was superior to conventional brazing and had no effect on the bond strength [30]. R. Rocha et al. presented that Co-Cr alloys welded with TIG showed a higher flexural strength than Nd:YAG laser-welded and non-welded samples [25]. S. Zangeneh et al. investigated the microstructural evolution of TIG-welded Co-28Cr-5Mo-0.3C alloy and concluded that the precipitation of M_23_C_6_ carbides at the nanoscale leads to a significant increase in hardness [38]. M. Sahami-Nejad et al. subjected the alloy Co-27Cr-5Mo-0.05C to TIG welding with different protective gas mixtures and investigated the microstructure, hardness and residual stress [26].

The aim of this work is to microscopically analyze and investigate the mechanical properties of six commercially available cobalt-chromium alloys used in dental practice that were welded with the TIG process in order to determine the quality of the TIG process as a technology for joining metallic dental materials and the suitability of the alloys for TIG welding.

## 2. Materials and Methods

Castings of six cobalt-chromium alloys from different manufacturers were used in this study (Table 1). Three alloys, VI—COMP (Austenal, Köln, Germany), Wirobond C (Bego, Bremen, Germany) and I—BOND NF (Interdent, Celje, Slovenia), are intended for the production of metal-ceramic works; two, Wisil M (Austenal, Köln, Germany) and I—MG (Interdent, Celje, Slovenia), are intended for denture frameworks, while one alloy, Brealloy F 400 (Bredent, Senden, Germany), is suitable for both purposes. The Brealloy F 400 is a cheap alloy, while the price of Wirobond C is very high. The prices of other alloys is a little higher than that of Brealloy F 400. All alloys differ in their mechanical and physical properties. The chemical compositions and properties of the alloys investigated, as stated by the manufacturers, are listed in Table 3 and Table 4.

The wax specimens of VI-COMP and Wisil M, whose dimensions are 32 mm × 10 mm × 1 mm, were modelled in a rubber mold with a measuring accuracy of 0.01 mm. They are embedded in phosphate investment material (Prestovest, Zlatarna Celje, Celje, Slovenia). The flask was preheated to 750 °C in a programmed furnace (ZC G8, Celje, Slovenia). These two alloys were cast in a centrifugal casting machine. The flasks were air-cooled according to the manufacturer’s instructions, and the castings were sandblasted with 110 µm diameter aluminum oxide particles (Sandblast Barth 322, Barth, Germany) to remove the residue of the investment. The channels made for the casting were removed with cutting discs, and the surface of the samples was treated with sandpaper and polished to a high gloss with a deerskin brush. A polishing machine CDG (Carlo di Georgio, Milan, Italy) was also used.

The other four alloys (I-BOND NF, I-MG, Wirobond C and Brealloy F 400) were modeled from non-transparent polymer investments (Interdent, Celje, Slovenia) with dimensions of 32 mm × 10 mm × 1 mm and processed on a Nonstop cutter (Silfradent, Santa Sofia, Italy). They were embedded in Intervest K+B speed investment material (Interdent, Celje, Slovenia) at 950 °C for 60 min. The alloys were inductively melted and cast in a pressure-vacuum casting machine (Nautilus, Bego, Bremen, Germany). The castings were also sandblasted with particles of 99.96% pure aluminum oxide with a diameter of 250 µm. Surface imperfections and oxides were removed electrolytically in an ELTROPOL electrolyte (Bego, Bremen, Germany) at 45 °C for 10 min until a high gloss was achieved.

Twenty-seven samples of each alloy were cast with the specified dimensions. In the next phase, six samples of each alloy were cut in half on an Accuto 2 cutter (Stuers, Rødovre; Denmark), and three samples served as a control group.

### 2.1. Joining of Samples

The cut samples were joined together using TIG. Welding using the TIG method was performed with a Primotec Phaser Mx1 unit (Hafner, Pforzheim, Germany) under argon to protect the surface of the specimens from oxidation and contamination and to improve arc stability [26]. Before joining, the cut surfaces were blasted with aluminium oxide particles with a diameter of 250 µm for 10 ms under a pressure of 4 bar in an Easy Blast sandblaster (Bego, Bremen, Germany) and then air-dried. The welding parameters were chosen in accordance with the manufacturer’s specifications. The welding parameters were as follows: operating voltage 20 V, current 30 A, argon flow 5 L/min, gas pressure 1 bar, pulse duration 18 ms; the tungsten electrode was used at an angle of 45–60 degrees, as it is consumed least in this way.

### 2.2. Microscopic Analysis of Welds

For the microscopic analysis of the welds, the appearance, the width, the homogeneity and the porosity of the welds were characterized with a light microscope Olympus GX51 with a CCD camera (Tokyo, Japan). The samples for metallographic analysis were prepared by embedding in Varidur 20, grinding, polishing and etching. For polishing, polishing cloths and pastes with diamond abrasives with a diameter of 3 µm and 1 µm were used. Lubricant blue (Stuers, Rodovre, Denmark) was also used. After polishing, the samples were electrolytically etched with a 10% chromic acid solution at a current of 100 mA for 30 s.

### 2.3. Mechanical Testing of the Samples

**Figure 1:**
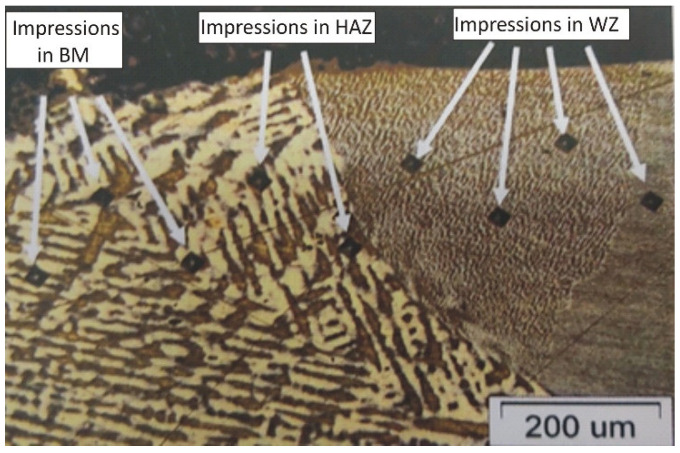
The scheme of the microhardness measurement.

Microhardness was measured on all samples at a load of 1.96 N (HV0.2), using a microhardness tester PMT-3. The measurement is carried out according to the Vickers method. The microhardness measurements were taken on each sample horizontally across the entire section, covering the base material (BM), the heat-affected zone (HAZ) and the weld zone (WZ). On each section, 10 impressions were made in a zigzag line, with a distance of 10 µm between each impression (Figure 1). Three series of measurements were carried out on each sample, and the mean values were calculated.

The flexural strength was determined on a mechanical testing machine (VEB Werkstoffprüfmaschinen, Berlin, Germany). The nominal test load was 400 kN, and the measuring range was between 0.1% and 100% of the nominal load. The tests were carried out on three unwelded and three welded specimens of the respective alloys. The specimens were loaded at three points. The distance between the supports was 24 mm, and the samples were placed so that the load was directly on the joint. The sample was loaded with a constant displacement of the mandrel of 5 mm/min. The test continued until the sample broke or slipped out of the fixture. During the test, the force and corresponding deflection of the sample were measured continuously. The flexural strength test resulted in flexural strength values of each sample and the deflection at which the sample broke or slipped.

The dynamic tests were carried out on three unwelded and three welded specimens of the respective alloys using the universal testing machine (LRX, Lloyd Instruments, Fareham, UK) with NEXYGEN™ 4.0 Software Package at room temperature. The distance between the supports was 24 mm, the mandrel diameter was 1 mm and the speed of the mandrel was 10 mm/min. Based on the maximum possible masticatory forces, the number of cycles was set at 1000. For each sample, it was recorded at which cycle it broke or that it survived all 1000 cycles without visible damage or fracture.

### 2.4. Statistical Analysis

For the description of flexural strength and the deflection, the mean value and the standard deviation are given. The factorial MANOVA was used to test the influence of the alloy (Vi-Comp, Wirobond C, I-Bond NF, Wisil M, I-MG and Brealloy F 400) and the group (type of welding: control group and TIG) on the flexural strength and deflection. The Tukey test was used for post-hoc multiple comparisons. The analysis was performed with the statistical package SAS on the Windows platform. All tests were performed with a significance level of α = 0.05.

## 3. Results

### 3.1. Microscopic Analysis

**Figure 2:**
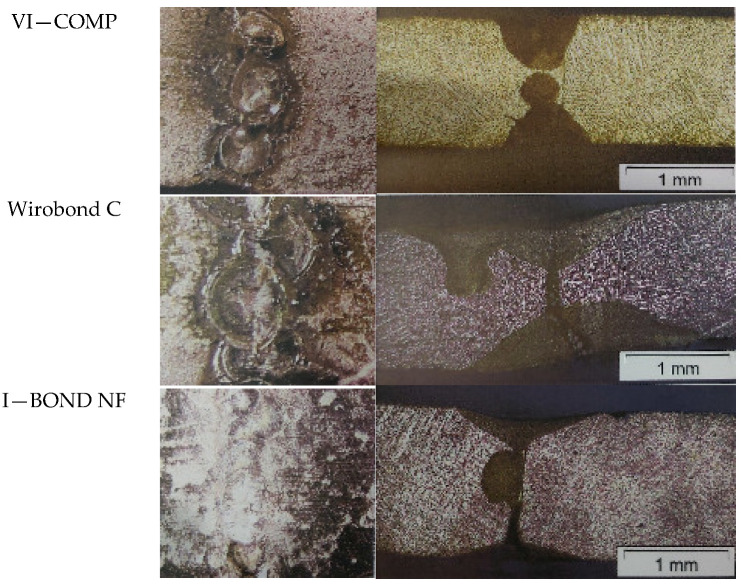

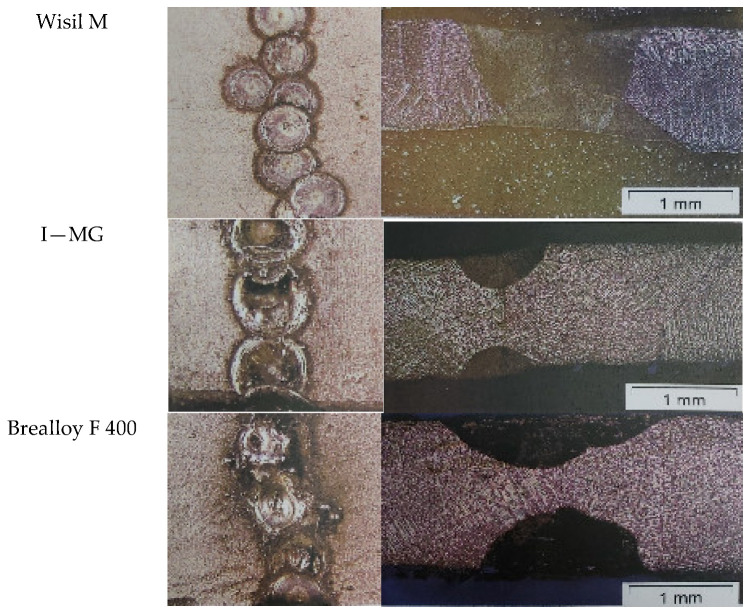
The appearance of the weld face and micrographs of all TIG-welded alloys.

The base material, the heat-affected zone and the weld were inspected and evaluated microscopically. The homogeneity of the weld, the grain size and shape, defects in the weld, i.e., weld notches, defects in the face and root of the weld, weldability, porosity and foreign inclusions were observed. The appearance of the weld face surface and the cross-sectional surface of all the investigated alloys are shown in Figure 2.

As can be seen in the micrograph, VI-COMP shows a messy, multi-layered weld. Porosity was observed in the form of small holes distributed along the edge of the weld. The non-perforated part of the weld is visible in a cross-section. Wirobond C shows a very unclean weld with unacceptable overlap of the welds and repeated welding in some places, as well as burrs in the form of sharp notches at the edges of the weld. Microscopic analysis revealed a weld that was not fused through with melted base material in the non-welded part of the joint. I—BOND NF has the neatest, most uniform weld that was ground after joining. The unwelded part is visible in the micrograph. The Wisil M sample shows a uniform joint with a reasonable overlap without macro irregularities and misalignment at the interface. However, a crack was observed, extending in the longitudinal direction of the weld. Joint I—MG is evaluated as a clean single-pass weld with inadequate overlap of the spot welds. Microscopic analysis shows a weld that is not through-welded. Brealloy F 400 exhibits an unclean multi-layer weld. Porosity is visible on the edges of the spot welds and indentations on the surface of the welds. Microscopic analysis also shows a weld that is not completely welded.

### 3.2. Results of Mechanical Testing

#### 3.2.1. Microhardness

**Figure 3:**
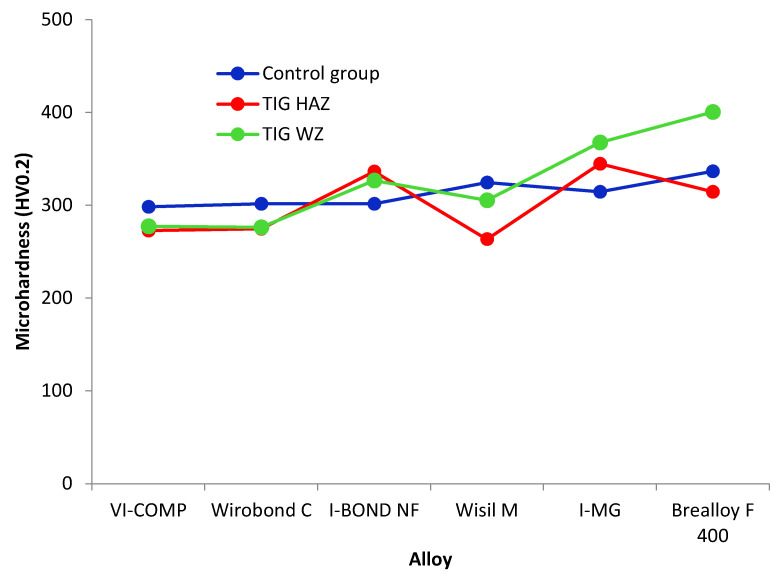
Microhardness (mean value) of the control group (blue line), the heat-affected zone (red line) and the weld zone (green line) of all test alloys.

To assess the joint quality, the microhardness in the area of the base material (BM), the heat-affected zone (HAZ) and the weld zone (WZ) is measured using the Vickers method. Table 5 and Figure 3 show the results of the statistical analysis of the microhardness measurements. These results show that the microhardness of the base material of the individual alloys is very similar and ranges between 298.3 and 336.6 HV0.2.

Comparing the values for the base materials of the individual alloys, it can be seen that the lowest microhardness was measured for the alloy VI-COMP and the highest for Brealloy F 400. When comparing the groups in terms of intended use, the base materials of the alloys for metal-ceramic works had lower microhardness values than the alloys for dental frameworks. Regarding the heat-affected zone of each alloy, Wisil M had the lowest microhardness, while the highest value was measured in the HAZ of I-MG. In the weld zone, the highest value of microhardness was measured for Brealloy F, and the lowest was measured for Wirobond C and VI-COMP. In general, similar to the base material, the heat-affected zones of alloys for metal-ceramic works had lower microhardness values than alloys for dental frameworks. The alloy for both purposes, Brealloy F 400, had the highest microhardness of all alloys in the weld zone.

#### 3.2.2. Flexural Strength

**Figure 4:**
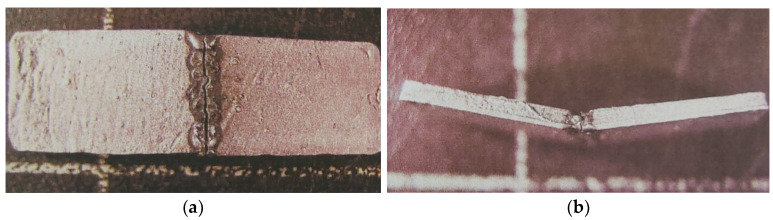
TIG weld of VI-COMP after flexural strength testing. (**a**) Weld face; (**b**) Side view of TIG weld.

All samples were tested for flexural strength. The appearance of the TIG-welded alloy VI-COMP after a three-point bending test is shown in Figure 4.

**Figure 5:**
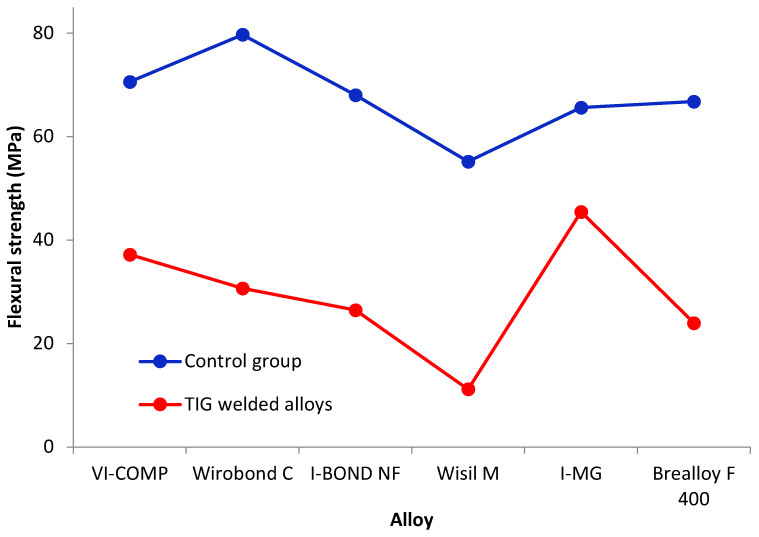
Flexural strength (mean value) of the control group (blue line) and TIG-welded alloys (red line).

**Figure 6:**
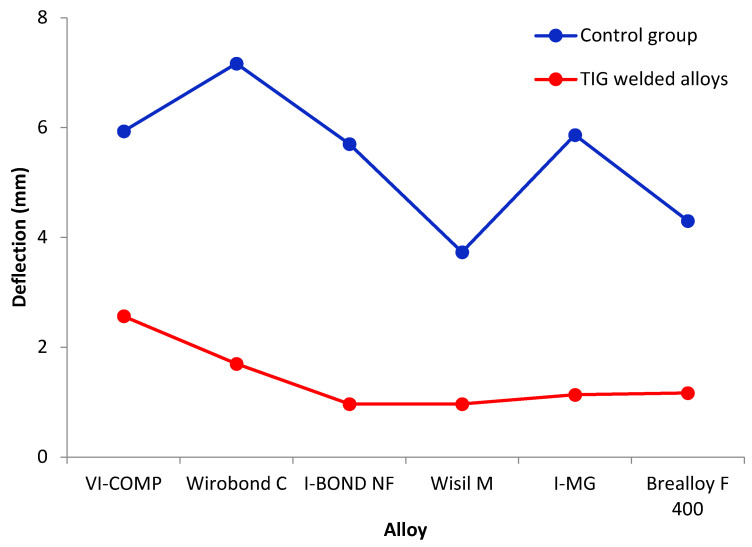
Deflection (mean value) of the control group (blue line) and TIG-welded alloys (red line).

Flexural strength tests resulted in force values and the deflection achieved for this force. The force that caused the weld or base material to break represents the maximum force that the material can withstand. The value of this force is used to calculate the flexural strength of the tested material samples. The results of the statistical analysis of the flexural strength measurements are shown in Table 6 and Figure 5. Table 7 and Figure 6 show the results for deflection.

In the first step, the influence of the welding type and the alloy on the flexural strength and the deflection was tested with the MANOVA test.

Table 8 shows that Wilks’ lambda is significant for both factors (group and alloy) and the interaction of the group and alloy (*p* < 0.05 for Wilks’ lambda). In the next step, the influence of the alloy and the type of welding on the flexural strength was tested with the ANOVA test. The results obtained are listed in Table 9.

Table 9 shows that the group (type of weld) and the type of alloy affect the flexural strength (*p* < 0.0001 and *p* = 0.0002; ANOVA test). Similarly, the interaction of the alloy and weld type is significant (*p* = 0.036; ANOVA test). Due to the significant interaction of the alloy and group, a comparison of all 12 groups was performed using the Tukey test, comparing only the alloys for each weld type and the weld types for each alloy separately (36 comparisons). The result of the comparison is shown in Table 10.

The flexural strength is significantly higher for all alloys in the control group than for the TIG-welded alloys, with the exception of I-MG, where no statistically significant difference was found between the control group and the TIG -welded alloys.

A comparison between the alloys in the control group showed that the flexural strength for Wisil M (55.2 MPa) was significantly lower than the flexural strength for Wirobond (79.7 MPa). There is no statistically significant difference between the other pairs.

For TIG-welded alloys, the flexural strength for I-MG (45.5 MPa) and VI-COMP (37.2 MPa) is significantly higher than the flexural strength for Wisil M (11.2 MPa). The flexural strength for I-MG (45.5 MPa) is significantly higher than the flexural strength for Brealloy F 400 (23.9 MPa). The comparison of the other alloy pairs showed no significant difference in flexural strength.

The analysis of the effect of the weld type and alloy on deflection showed that the effects of the weld type and alloy type were significant (*p* < 0.0001 and *p* = 0.0135 ANOVA test; Table 11), while the interaction of the weld type and alloy is not significant (*p* = 0.20; ANOVA test).

The influence of the welding type on the deflection is shown in Table 12. In the control group, the deflection is significantly higher than that in the TIG -welded alloys (on average, 5.5 mm compared to 1.4 mm).

A comparison of the deflections for the different alloys is shown in Table 12. Although the ANOVA test showed that there was a difference between the alloys (Table 11), the Tukey test did not show which alloys differed from each other.

#### 3.2.3. Dynamic Testing

**Figure 7:**
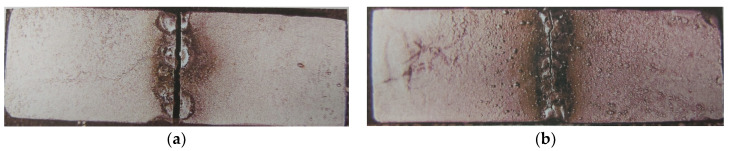
Macrographs of TIG-welded samples after dynamic testing. (**a**) Wirobond C; (**b**) I-BOND NF.

The resistance of the weld samples to dynamic loads was also tested. The tests were carried out on a dynamic mechanical machine for 1000 cycles. Figure 7 shows macrographs of samples of TIG-welded Wirobond C, which survived the lowest number of cycles, and of I-BOND NF, which survived the highest number (1000) of cycles.

Since there was a difference in the dynamic load withstand between the unwelded and welded alloys, statistics were compiled for these two groups. The influence of the weld type on the resistance of the welds to dynamic loads is shown in Table 13. The results of the dynamic tests show that the samples of the control groups of all alloys withstood 1000 cycles. The resistance of the control group is significantly higher than that of the TIG-welded alloys (on average, 1000 cycles compared to 648.9 cycles).

A comparison of the weld resistance under dynamic loading for the different alloys is shown in Table 13. The ANOVA test showed that the mean values do not differ significantly at the 0.05 level.

## 4. Discussion

In everyday life, it often happens that various prosthetic works made of cobalt-chromium alloys break and have to be repaired and rejoined. Dental technicians who use laser and TIG welding techniques are responsible for repairing cast alloys [39,40]. In this study, to determine the functional durability of prosthetic work, six specimens of a Co-Cr alloy were welded using TIG. During welding, the specimens were held in the hands, so there is a possibility that the specimens may move, the hands may shake and human error may occur, resulting in uneven weld spots, i.e., overlapping welds. To join the specimens as well as possible, so-called “jokers” are now used. These are holders that are fixed and serve to better control so that the specimens do not move. This results in joints with fewer cracks and optimal overlap [31].

Mosch proved the connection between the duration of the welding pulse and the formation of cracks when welding Co-Cr alloys. If the pulse lasts only a short time, e.g., 3 ms, there is a higher probability of microcracks, because the alloy cools much faster around the welding spot than around the rest of the specimen. Due to the contraction of the material during cooling, cracks appear, and if the welding continues regardless of the crack, the material will break [31]. Our results prove that the occurrence of cracks and fractures does not only depend on the welding time, because the time of a pulse in TIG was 10 ms, which is significantly longer than that in the study described by Mosch. This also indicates the influence of other factors. Matsuda demonstrated in his research that the occurrence of a crack depends on whether or not stresses are present in the base material, and if this is the case, the crack is longer [41]. In general, the mechanism of cracking and fracture formation is extremely complex and depends on various physicochemical properties of the alloy, different directions and magnitudes of stress, thermal changes and humidity conditions in the oral cavity and, of course, the general health of the patient.

The mechanical properties of welded joints depend on many factors, one of the most important being the welding technique. It is necessary to control the weld of the material macroscopically and microscopically at the same time [42]. In this study, longitudinal cracks were the most common, while radial cracks were the least common. Radial cracks are the result of stresses within the weld, and longitudinal cracks are the result of bending of the parts during joining.

In this study, similar values for the microhardness of the base material were measured for the alloys, with the highest value being for Brealloy F 400 and the lowest being for VI-COMP. Of all the alloys, Brealloy F 400 also had the highest microhardness in the weld zone. Minor differences were found in the microhardness of the heat-affected zone. In contrast to the results in [43], where Górka et al. showed that TIG joints of Inconel joints were characterized by a significantly higher hardness of the weld area and lower hardness of the HAZ and the base material, this was shown in this study for Brealloy F 400 and I-MG alloys. In contrast, the weld zone of VI-COMP showed lower microhardness than the heat-affected zone and the base material. The differences in microhardness values are the result of microstructural changes during welding, the chemical composition of the material, different grain sizes and the formation of precipitates. Roggensach [44] also concluded that the differences in microhardness of the weld material and the base material are actually a result of the grain size and microstructure that occur during the cooling of the alloy.

When testing the flexural strength for the same deflection, the specimens VI-COMP, Wirobond C and I—BOND NF were loaded with a higher force. For I-MG and Brealloy F-400, the force values were higher when TIG was used. The highest stress was calculated for I-MG welded with TIG, as the joint was optimally designed considering the direction and overlap of the welds. The widest range of deflection and stress values was obtained with the Wirobond C material.

Topham et al. demonstrated in their study that a joint welded on both sides is much stronger than one welded on one side, regardless of the number of spot welds. It was also found that the percentage of elongation correlated with the force applied to the specimen [45]. In this study, the specimens were welded on both sides, but some TIG joints cracked due to dynamic loading. At the same time, it was found that the cracked samples were improperly welded, which is certainly one of the factors for the lower durability of the sample. The welding depth of the cracked specimens was 0.3 mm. The dynamic tests with a dynamic mechanical machine on all six castings gave different results. All control specimens survived all 1000 cycles without cracking. Of the welded specimens, those from I-BOND NF and Wisil M also survived all 1000 cycles without cracking.

Castings of base alloys crystallize in dendritic form due to the rate of cooling and the proportion of several constituents with different melting points, as well as the lower chemical affinity between the individual metals. The dendrites are partially melted due to the welding speed, and their size is a consequence of the cooling rate, so they are not very homogeneous in their structure [46]. However, it is also possible to homogenize the casting by subsequent heat treatment, but this takes several hours. The inhomogeneity of cobalt-chromium-based alloy castings is compensated for by the property of surface passivation. Argon creates a protective atmosphere around the weld and protects the molten metal from undesirable reactions with oxygen, nitrogen and other gases that are released during welding and lead to discoloration of the metal, a conditional increase in hardness and increased brittleness of the entire structure. Watanabe et al. [47] concluded that for joints welded under argon shielding gas, a lower force leads to the fracture and elongation of the specimens. In contrast, Baba et al. investigated that joining under argon shielding gas has no influence on the flexural strength and fracture resistance of Co-Cr alloys [48]. In this study, all specimens were welded with TIG under inert gas, with argon pressure of 1 bar.

In the flexural strength test, the deflection and strength of the metal-ceramic castings were greater than those of the prosthetic frameworks. In their study, Rocha et al. [25] were convinced that TIG-welded Co-Cr joints had higher flexural strength values than unwelded samples. This was not confirmed in this study. In fact, the unwelded samples of the alloys showed a higher flexural strength than those in the heat-affected zone and weld zone. The TIG parameters were 10 V, 15–20 A and 12 ms. In another study by Watanabe et al. [49], it was shown that subsequent heat treatment increases the strength of welds between alloys, as precipitation and hardness are correlated.

The results of the Vickers hardness measurements show that the TIG welding had no significant effect on the hardness of the tested alloys. According to the present results, TIG welding had the greatest influence on the hardness of alloy I—MG and the least influence on the hardness of Brealloy F 400. The hardness of the welded alloys VI—COMP, Wirobond C and Wisil M decreased, while the hardness of I—BOND NF, I—MG and Brealloy F 400 increased. The reason for the decrease in hardness could be improperly performed welding work, leading to inadequate welds and even cracks. Considering that Co-Cr alloys are hardened primarily by carbide formation [21] and that the welding work was carried out correctly, an increase in the hardness of I—BOND NF, I—MG and Brealloy F 400 is to be expected. Differences in the flexural strength values could be explained by possible irregularities under the surface such as microporosity, which did not affect the measured hardness and were not visible microscopically. These differences can be attributed to pores, cracks and other defects that occurred on the joint during the welding process. Regarding the deflection values for TIG, the welded Wisil M showed the least decrease in deflection. The reason for this is to be found in the quality of the weld, which was confirmed by withstanding the maximum number of cycles under dynamic loading. Taking into account all the results obtained, the alloys TIG—welded I—BOND NF and Wisil M showed the cleanest and most uniform weld and, accordingly, satisfactory mechanical properties, highlighting that they withstood the maximum number of cycles under dynamic loading.

### Limitations of the Study

Despite all efforts, the study has certain limitations. For example, the quality of the welds, which affects the mechanical properties, is highly dependent on the skill and experience of the operator. Moreover, the process is not easy to automate. To date, there is no consensus for the selection of optimal welding parameters based on the material properties and geometry of a particular restoration. As mechanical properties were determined in vitro in this work, future work should simulate in vivo conditions and conduct clinical trials.

## 5. Conclusions

The results of this study showed that the mechanical properties were not significantly affected by TIG welding. Furthermore, this study showed the relationship between the basic mechanical properties of Co-Cr dental alloys and the quality of welding performance. Therefore, the person performing the welding work should have good eyesight and hand-eye coordination and be skilled in using hands. It was found that the differences in the results were due to the welding parameters and the skill and precision of the dental technician. The differences between the specimens are in the shape, size and appearance of the welds, resulting in differently measured mechanical properties, which also affects the quality of the prosthetic work. It has been shown that the optimal fixation of the specimens is necessary to find parameters that ensure a satisfactory weld and avoid irregularities during the welding process itself. From an economic point of view, i.e., when comparing the prices of the individual alloys and based on the results obtained from the group of alloys for metal-ceramic works, the alloy I—BOND NF can be recommended as an alloy that can be welded with TIG, while from the group of alloys for partial dentures, the alloy Wisil M is recommended. In contrast, the alloy Wirobond C is not recommended for TIG welding in dental applications.

## Figures and Tables

**Table 1:** A list of the different dental alloys and their mechanical properties [3,4,5,6,7,8,9,10,11,12,13,14,15,16,17,18,19,20].

Alloy Type	Modulus of Elasticity, GPa	Yield Strength, MPa	Tensile Strength, MPa	Elongation, %	Vickers Hardness
Au-based	86–135	240–690	405–790	10.0–40.0	125–300
Pd-based	119–141	450–795	1002–1259	2.7–25.1	200–245
Ti-based	44–113	170–1060	240–1100	4–26.7	116–500
Ni-Cr	47–440	375–753	720–862	4.4–21.5	203–430
Co-Cr	198–234	500–510	620–660	5.0–12.0	360–385
Cu-Al	64–123	50–508	189–595	0.1–47.4	50–306
Ni-Ti	50–120	138–379	690–1380	3.0–60.0	170–660
Ag-Pd	80–106	300–475	500–610	5.0–18.0	150–240

**Table 2:** The classification and mechanical properties of the commonly used Co-Cr dental alloys [21,22,23,24].

Composition Type	Application Type	Tradename	Modulus of Elasticity, GPa	Yield Strength, MPa	Tensile Strength, MPa	Elongation, %	Vickers Hardness
Co-Cr-Mo	partial dentures	Wironium	220	640	940	10	360
		Wironium plus	220	700	1000	13	340
		Wironit	185	615	895	10	360
		Suprachrome	200	600	N/A *	9	410
		Vitallium	200	680	960	10	395
		Biosil	209	410	N/A *	N/A *	335
Co-Cr-W	metal-ceramic	StarLoy C	255	520	800	10	N/A *
		Remanium Star CL	230	600	1100	32.5	N/A *
Co-Cr-Nb	metal-ceramic	Magnum Lucens	230	490	600	2.0	324

N/A *—not applicable.

**Table 3:** Chemical composition of commercially available alloys.

Tradename	Composition (wt.%)											
	Co	Cr	Mo	Mn	Si	N	C	Nb	W	Fe	Ni	Ce
VI—COMP	61.0	32.0	5.5	0.7	0.7	-	-	-	-	-	-	-
Wirobond C	61.0	26.0	6.0	-	1.0	-	0.02	-	5.0	0.5	-	0.5
I—BOND NF	63.0	24.0	3.0	-	1.0	-	-	1	8.0	-	-	-
Wisil M	63.1	28.0	6.0	1.0	0.8	-	0.50	-	0.6	-	-	-
I—MG	62.5	29.5	5.5	0.6	1.4	0.2	0.30	-	-	-	-	-
Brealloy F 400	64.7	29.0	5.0	0.4	0.5	-	0.40	-	-	-	-	-

**Table 4:** Properties of commercially available alloys studied.

Tradename	Density, g/cm^3^	E, MPa	HV10	Elongation, %	Melting Interval, °C
VI—COMP	8.3	200	320	14	1270–1345
Wirobond C	8.5	210	310	6	1270–1380
I—BOND NF	8.3	210	285	10	1304–1369
Wisil M	8.4	230	410	5	1335–1365
I—MG	8.2	220	365	7.5	1295–1345
Brealloy F 400	8.4	220	400	10	1320

**Table 5:** Microhardness (HV0.2): sample, mean and standard deviation (SD).

Group	Alloy	N	Mean	SD
	VI—COMP	3	298.3	5.8
	Wirobond C	3	301.5	4.9
Control	I—BOND NF	3	301.4	8.2
	Wisil M	3	324.5	9.4
	I—MG	3	314.5	6.2
	Brealloy F 400	3	336.6	8.1
TIG HAZ	VI—COMP	3	272.6	28.3
	Wirobond C	3	274.5	1.85
	I—BOND NF	3	336.4	10.9
	Wisil M	3	263.5	2.6
	I—MG	3	344.7	1.1
	Brealloy F 400	3	314.5	5.9
	VI—COMP	3	277.2	2.1
	Wirobond C	3	276.2	3.8
TIG WZ	I—BOND NF	3	326.5	3.7
	Wisil M	3	305.4	3.8
	I—MG	3	367.6	41.8
	Brealloy F 400	3	400.5	10.9

**Table 6:** Flexural strength (MPa): sample size, mean and standard deviation (SD).

Group	Alloy	N	Mean	SD
	VI—COMP	3	70.6	8.2
	Wirobond C	3	79.7	7.6
Control	I—BOND NF	3	68.0	10.7
	Wisil M	3	55.2	2.5
	I—MG	3	65.6	8.9
	Brealloy F 400	3	66.8	2.8
	VI—COMP	3	37.2	13.0
	Wirobond C	3	30.7	4.9
TIG-welded	I—BOND NF	3	26.4	4.3
	Wisil M	3	11.2	4.6
	I—MG	3	45.5	9.8
	Brealloy F 400	3	23.9	2.5

**Table 7:** Deflection (mm): sample size, mean and standard deviation (SD).

Group	Alloy	N	Mean	SD
	VI—COMP	3	5.9	1.1
	Wirobond C	3	7.2	0.3
Control	I—BOND NF	3	5.7	0.2
	Wisil M	3	3.7	0.2
	I—MG	3	5.9	0.7
	Brealloy F 400	3	4.3	0.6
	VI—COMP	3	2.6	3.2
	Wirobond C	3	1.7	0.8
TIG-welded	I—BOND NF	3	1.0	0.2
	Wisil M	3	1.0	0.2
	I—MG	3	1.1	0.1
	Brealloy F 400	3	1.2	0.1

**Table 8:** Results for the MANOVA test.

Factor	Wilks’ Lambda	*p*
Group	0.08	<0.0001
Alloy	0.30	0.0008
Interaction	0.44	0.02

**Table 9:** ANOVA test for flexural strength.

Source of Variation	DF	SS	MSS	F	*p*
Group	1	13,336.4	13,336.4	239.82	<0.0001
Alloy	5	2217.2	443.4	7.97	0.0002
Interaction	5	799.1	159.8	2.87	0.036
Error	24	1334.6	55.6		
Total	35	17,687.4			

**Table 10:** Comparison of flexural strength depending on the alloy and the type of welding.

	Control Group	TIG-Welded Alloys
Alloy	Mean	Mean
VI—COMP	70.6 ^ab^	37.2 ^ce^
Wirobond C	79.7 ^a^	30.7 ^cde^
I—BOND NF	68.0 ^ab^	26.4 ^cde^
Wisil M	55.2 ^b^	11.2 ^d^
I—MG	65.6 ^abA^	45.5 ^cA^
Brealloy F 400	66.8 ^ab^	23.9 ^de^

^a,b,c,d,e^—there is no significant difference between the alloys with the same letter (Tukey test). ^A^—there is no significant difference between groups with the same letter (Tukey test).

**Table 11:** ANOVA table for deflection.

Source of Variation	DF	SS	MSS	F	*p*
Group	1	146.4	146.4	133.00	<0.0001
Alloy	5	20.1	4.0	3.65	0.0135
Interaction	5	8.8	1.8	1.61	0.20
Error	24	26.4	1.1		
Total	35	201.7			

**Table 12:** Comparison of the deflection (mm) depending on the type of weld and the type of alloy.

Group/Alloy	Mean
Control group	5.5
TIG-welded alloys	1.4
VI—COMP	4.3 ^a^
Wirobond C	4.4 ^a^
I—BOND NF	3.3 ^a^
Wisil M	2.4 ^a^
I—MG	3.5 ^a^
Brealloy F 400	2.7 ^a^

^a^—there is no significant difference between alloys with the same letter (Tukey test).

**Table 13:** Comparison of weld resistance to dynamic loading (number of cycles) depending on the type of weld and the type of alloy.

Group/Alloy	Mean
Control group	1000
TIG-welded alloys	648.9
VI—COMP	368.0
Wirobond C	329.3
I—BOND NF	1000.0
Wisil M	1000.0
I—MG	528.7
Brealloy F 400	667.3

## Data Availability

Not applicable.

## References

[1] Matković T., Slokar L.J., Matković P. (2006). Structure and properties of biomedical Co-Cr-Ti alloys. J. Alloys Compd..

[2] Carek A. (2005). Analysis of Base Alloys Solder Joints Depend on Different Welding Technology. Master’s Thesis.

[3] Slokar L.J., Pranjić J., Carek A. (2017). Metallic Materials for Use in Dentistry. Holist. Approach Environ..

[4] Yu J.-M., Kang S.-Y., Lee J.-S., Jeong H.-S., Lee S.-Y. (2021). Mechanical Properties of Dental Alloys According to Manufacturing Process. Materials.

[5] Papazoglou E., Wu Q., Brantley W.A., Mitchell J.C., Meyrick G. (1999). Mechanical properties of dendritic Pd-Cu-Ga dental alloys. Cells Mater..

[6] Anusavice K.J., Shen C., Rawls H.R. (2013). Phillips’ Science of Dental Materials.

[7] Nicholson J.W. (2020). Titanium Alloys for Dental Implants: A Review. Prosthesis.

[8] Shingade A., Dhatrak P. (2021). Biomaterials used in dental applications to improve success rate of implantation: A review. AIP Conf. Proc..

[9] Hoque M.E., Showva N.-N., Ahmed M., Rashid A.B., Sadique S.E., El-Bialy T., Xu H. (2022). Titanium and titanium alloys in dentistry: Current trends, recent developments, and future prospects. Heliyon.

[10] Slokar L.J., Živko-Babić J., Matković P. Evaluation of mechanical properties of titanium-based alloy for use in dentistry. Proceedings of the 23rd International Conference on Metallurgy and Materials METAL 2014.

[11] Liang S.X., Feng X.J., Yin L.X., Liu X.Y., Ma M.Z., Liu R.P. (2016). Development of a new β Ti alloy with low modulus and favorable plasticity for implant material. Mater. Sci. Eng. C.

[12] Correa D.R.N., Vicente F.B., Donato T.A.G., Arana-Chavez V.E., Buzalaf M.A.R., Grandini C.R. (2014). The effect of the solute on the structure, selected mechanical properties, and biocompatibility of Ti-Zr system alloys for dental applications. Mater. Sci. Eng. C.

[13] Thompson S.A. (2000). An overview of nickel-titanium alloys used in dentistry. Int. Endod. J..

[14] Močnik P., Kosec T. (2021). A critical appraisal of the use and properties of nickel–titanium dental alloys. Materials.

[15] Machio C., Mathabathe M.N., Bolokang A.S. (2020). A comparison of the microstructures, thermal and mechanical properties of pressed and sintered Ti–Cu, Ti–Ni and Ti–Cu–Ni alloys intended for dental applications. J. Alloys Compd..

[16] Yoneyama T., Doi H., Hamanaka H. (1992). Influence of Composition and Purity on Tensile Properties of Ni-Ti Alloy Castings. Dent. Mater. J..

[17] Dobrzański L.A., Dobrzański L.B., Dobrzańska-Danikiewicz A.D., Dobrzańska J. (2022). Nitinol Type Alloys General Characteristics and Applications in Endodontics. Processes.

[18] Rittapai A., Urapepon S., Kajornchaiyakul J., Harniratisai C. (2014). Properties of experimental copper-aluminium-nickel alloys for dental post-and-core applications. J. Adv. Prosthodont..

[19] Achitei D.C., Baltatu M.S., Vizureanu P., Sandu A.V., Benchea M., Istrate B. (2022). Ni-Cr Alloys Assessment for Dental Implants Suitability. Appl. Sci..

[20] Yang K.R., Hanawa T., Kwon T.Y., Min B.K., Hong M.H. (2021). Mechanical property comparison of Ni–Cr–Mo alloys fabricated via one conventional and two new digital manufacturing techniques. Appl. Sci..

[21] Al Jabbari Y.S. (2014). Physico-mechanical properties and prosthodontic applications of Co-Cr dental alloys: A review of the literature. J. Adv. Prosthodont..

[22] Dolgov N.A., Dikova T., Dzhendov D., Pavlova D., Simov M. Mechanical properties of dental Co-Cr alloys fabricated via casting and selective laser melting. Proceedings of the II International Scientific-Technical Conference “Innovations in Engineering” 2016.

[23] Vaicelyte A., Janssen C., Le Borgne M., Grosgogeat B. (2020). Cobalt–Chromium Dental Alloys: Metal Exposures, Toxicological Risks, CMR Classification, and EU Regulatory Framework. Crystals.

[24] Kim H.R., Jang S.-H., Kim Y.K., Son J.S., Min B.K., Kim K.-H., Kwon T.-Y. (2016). Microstructures and mechanical properties of Co-Cr dental alloys fabricated by three CAD/CAM-based processing techniques. Materials.

[25] Rocha R., Pinheiro A.L.B., Villaverde A.B. (2006). Flexural strength of pure Ti, Ni-Cr and Co-Cr alloys submitted to Nd:YAG laser or TIG welding. Braz. Dent. J..

[26] Sahami-Nejad M., Lashgari H.R., Zangeneh S., Kong C. (2019). Determination of residual stress on TIG-treated surface via nanoindentation technique in Co-Cr-Mo-C alloy. Surf. Coat. Technol..

[27] Voruganti K. (2008). Dental materials: Properties and manipulation (9th edition). Br. Dent. J..

[28] Berthod P., Bouaraba M., Cai J. (2023). Influence of the Chromium Content on the Characteristics of the Matrix, the Tantalum Carbides Population, and the Hardness of Cast Co(Cr)-0.4C-6Ta Alloys. Micro.

[29] Carek A., Babic J.Z., Schauperl Z., Badel T. (2011). Mechanical Properties of Co-Cr Alloys for Metal Base Framework. Int. J. Prosthodont. Restor. Dent..

[30] Matos A.O., Branco C.d.C.C.C., Klautau E.B., Alves B.P. (2017). Comparative analysis of ceramic flexural strength in co-cr and ni-cr alloys joined by TIG welding and conventional brazing. Brazilian J. Oral Sci..

[31] Mosch M.H.J., Hoffmann A. (2004). Lightning in a Bottle. Dent. Dialogue.

[32] Bock J.J., Fraenzel W., Bailly J., Gernhardt C.R., Fuhrmann R.A.W. (2008). Influence of different brazing and welding methods on tensile strength and microhardness of orthodontic stainless steel wire. Eur. J. Orthod..

[33] Da Silveira-Júnior C.D., de Castro M.G., Davi L.R., das Neves F.D., Kovacevic R. (2012). Welding Techniques in Dentistry. Welding Processes.

[34] Zupančič R., Legat A., Funduk N. (2007). Electrochemical and mechanical properties of cobalt-chromium dental alloy joints. Mater. Tehnol..

[35] De Castro G.C., de Araújo C.A., Mesquita M.F., Consani R.L.X., Nóbilo M.A.D.A. (2013). Stress distribution in Co-Cr implant frameworks after laser or TIG welding. Braz. Dent. J..

[36] Carek A., Schauperl Z., Jakovac M. (2007). Macroscopic Analysis of Co-Cr Base Alloys Joints. Acta Stomatol. Croat..

[37] De Castro Silverio M.G., Raposo L.H.A., Lopes R.T. (2021). Evaluation of X-shaped welded joints with Co-Cr alloy under different welding parameters: Analysis by micro-CT and flexural strength. Res. Soc. Dev..

[38] Zangeneh S., Lashgari H.R., Lopez H.F., Farahani H.K. (2017). Microstructural characterization of TIG surface treating in Co-Cr-Mo-C alloy. Mater. Charact..

[39] Mosch M.H.J., Hoffmann A. (2004). Lightening in a Bottle State of the art joining techniques in dental technology Part 2 in a series. Dent. Dialogue.

[40] Nowacki J., Rybicki P. (2005). The influence of welding heat input on submerged arc welded duplex steel joints imperfections. J. Mater. Process. Technol..

[41] Matsuda F., Ueyama T. (1993). Solidification crack susceptibility of laser weld metal in 0.2c-ni-cr-mo steels: Effects of bead configuration and s and p contents. Weld. Int..

[42] Toyoda M., Mochizuki M. (2004). Control of mechanical properties in structural steel welds by numerical simulation of coupling among temperature, microstructure, and macro-mechanics. Sci. Technol. Adv. Mater..

[43] Górka J., Jamrozik W., Kiel-Jamrozik M. (2023). The effect of TIG welding on the structure and hardness of butt joints made of Inconel 718. Heliyon.

[44] Roggensack M., Walter M.H., Böning K.W. (1993). Studies on laser- and plasma-welded titanium. Dent. Mater..

[45] Topham T., Scott D., Watanabe I., Baba N., Okabe T. Mechanical Properties of Laser-welded Dental Casting Alloys. Proceedings of the 2003 AADR/CADR Annual Meeting.

[46] Baba N., Watanabe I., Liu J., Atsuta M. (2004). Mechanical Strength of Laser-Welded Cobalt-Chromium Alloy. J. Biomed. Mater. Res.—Part B Appl. Biomater..

[47] Watanabe I., Topham D.S. (2006). Laser welding of cast titanium and dental alloys using argon shielding. J. Prosthodont..

[48] Baba N., Watanabe I., Tanaka Y., Hisatsune K., Atsuta M. (2005). Joint Properties of Cast Fe-Pt Magnetic AlloycLaser-welded to Co-Cr Alloy. Dent. Mater. J..

[49] Watanabe I., Benson A.P., Nguyen K. (2005). Effect of heat treatment on joint properties of laser-welded Ag-Au-Cu-Pd and Co-Cr alloys. J. Prosthodont..

